# Endovascular Management of a Large Femoral Pseudoaneurysm: A Case Report and Literary Review

**DOI:** 10.7759/cureus.23045

**Published:** 2022-03-10

**Authors:** Karlbuto Alexandre

**Affiliations:** 1 Department of Surgery, Detroit Medical Center (DMC) Sinai-Grace Hospital, Detroit, USA

**Keywords:** pseudoaneurysm, percutaneous coronary intervention complications, percutaneous endovascular repair, percutaneous, general and vascular surgery

## Abstract

Femoral artery pseudoaneurysms have an increased incidence over the past few years due to the rise in percutaneous catheterization and so have the potential treatment options. Ultrasound-guided thrombin injection has been strongly studied, and data have shown its efficacy, safety, and superiority to ultrasound-guided compression therapy as well as open surgical repair; however, a less well-studied approach that appears to be burgeoning is endovascular stent repair. Many small studies and case reports have shown this option to be not only effective but also safe and might be the treatment option of choice in patients who are deemed high risk for surgical intervention or with complicated anatomical considerations at the site of injury. In this case report, we describe a 71-year-old man with an expanding right groin hematoma which was discovered to be a right superficial femoral artery pseudoaneurysm with a venous fistula connection to the common femoral vein. Due to the patient’s venous fistula component, high surgical risk from substantial comorbidities, and large pseudoaneurysm size with a wide pseudoaneurysm neck, thrombin injection, compression therapy, and open surgical repair were ruled out as potential treatments; therefore, endovascular stent repair was performed. The procedure was successful, as was the patient’s postoperative period. This case report and literary review can support and further validate the usage of endovascular stent repair to treat femoral artery pseudoaneurysms.

## Introduction

Over the past decade, the usage of percutaneous endovascular catheterization procedures for both diagnostic and interventional treatment options has increased substantially [1,2]. Inadvertently, so has the incidence of femoral artery pseudoaneurysms [1,2]. A pseudoaneurysm is like an aneurysm except for the fact that it is bounded by less than all three layers of the blood vessel wall, and it can even be encapsulated by either the surrounding tissue or a thin fibrous capsule around the vessel wall [1-4]. It is not contained within all three layers of the blood vessel wall unlike true aneurysms [1-4]. Currently, there are three main treatment options for patients who have femoral artery pseudoaneurysms. First, there is the use of ultrasound-guided thrombin injection which is the gold standard treatment; second, there is ultrasound-guided compression if thrombin injections failed; and third, there is open surgical repair [4-9]. However, a very efficient but yet underused treatment has been endovascular stent repair [9-20]. Many studies have confirmed its efficiency and safety; however, it still remains unused by vascular surgeons for pseudoaneurysm repair [9-20]. More specifically, this technique has been shown to be very effective in patients with complex anatomy with comorbidities, for which these patients are at a high surgical risk [13-20]. Here, we present a 71-year-old male who is at high risk for surgical intervention who presents with a large right superficial femoral artery pseudoaneurysm with venous fistula connection to the common femoral vein which was successfully repaired using endovascular stenting.

## Case presentation

**Figure 1:**
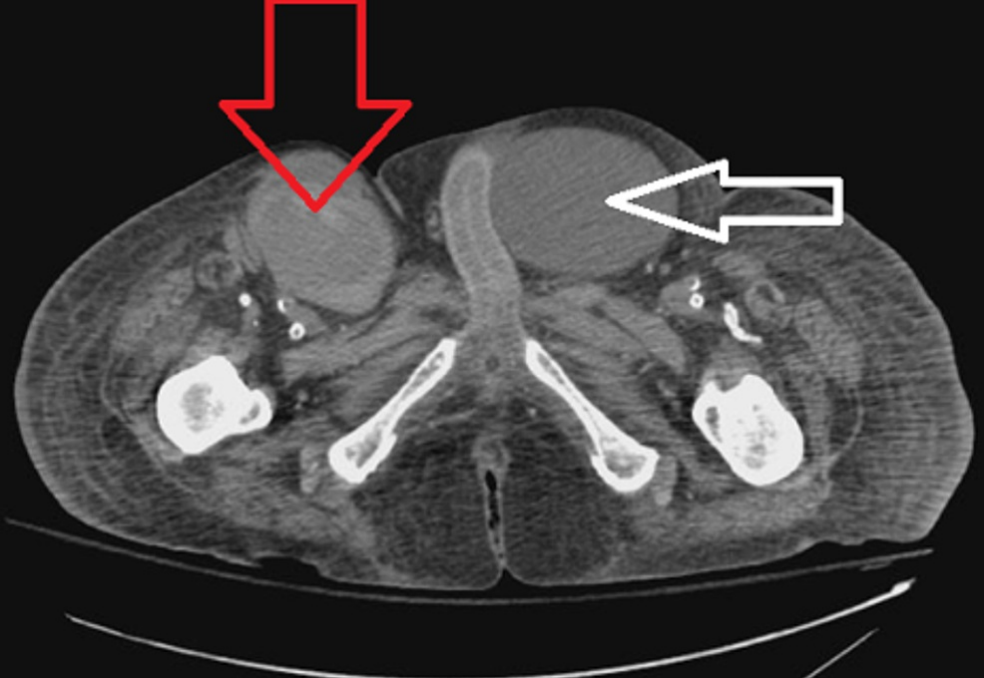
Axial CT scan without IV contrast. Red arrow is pointing at the right superficial femoral artery pseudoaneurysm. White arrow is pointing at a large inguinal hernia that was discovered concurrently.

**Figure 2:**
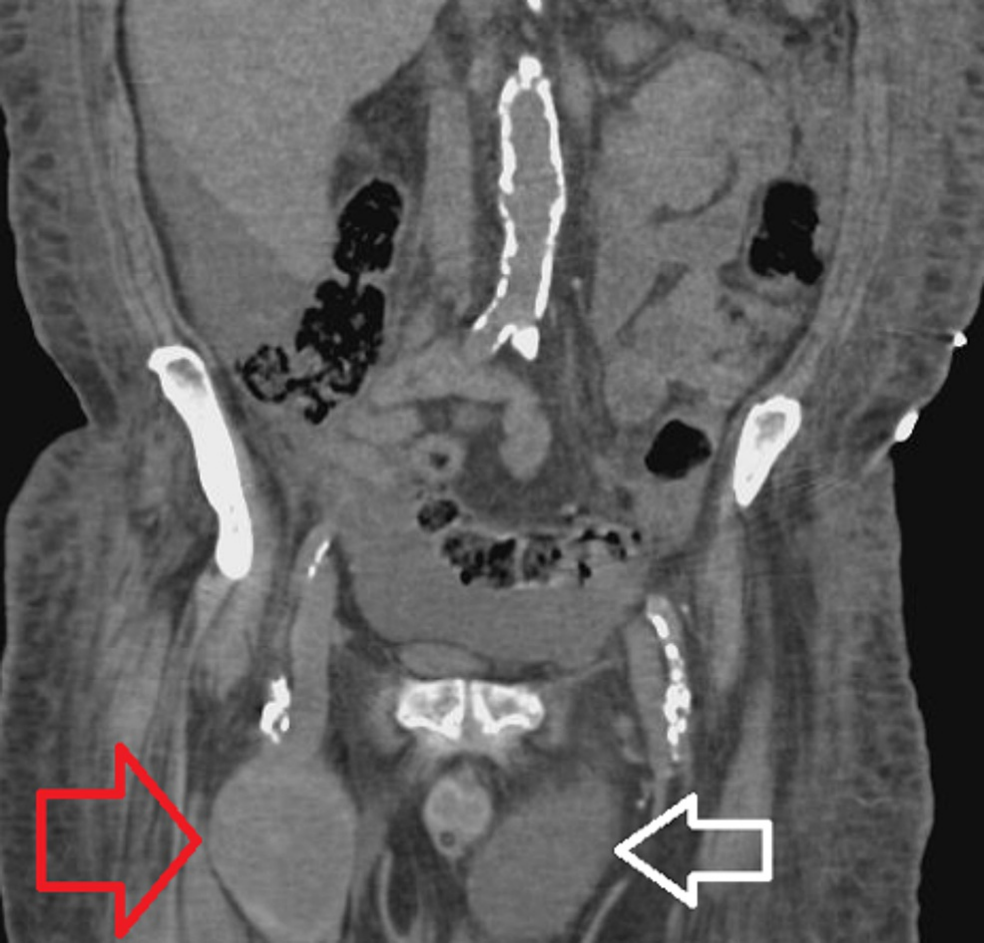
Coronal CT scan without IV contrast. Red arrow showing the large superficial femoral pseudoaneurysm. White arrow showing the large left inguinal hernia.

**Figure 3:**
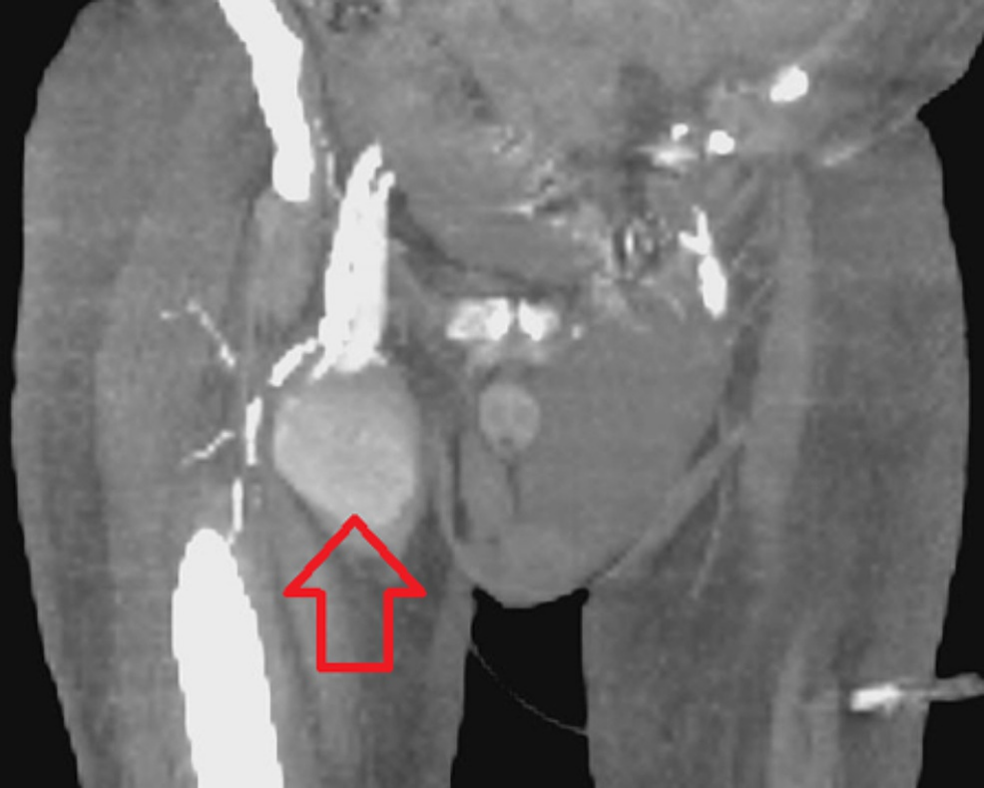
CT angiography of right lower extremity. Red arrow showing large right superficial femoral artery pseudoaneurysm.

A 71-year-old male with a significant past medical history of congestive heart failure, pulmonary hypertension, and end-stage renal disease with a tracheostomy presented to the emergency room from his nursing home facility for atrial fibrillation with a rapid ventricular rate. During his course of treatment, the patient developed increasing right groin pain after three days of hospitalization, and a noncontrast CT scan showed possible right groin hematoma, measuring 7.5 x 5.4 cm extending to the right femoral vein and artery as shown in Figures 1, 2. Subsequent computed tomography angiography (CTA) results showed a right superficial femoral artery pseudoaneurysm, measuring 7.8 x 5.8 x 11 cm, with a 2-3 mm very thin neck as shown in Figure 3. In addition, he also has right arm arteriovenous graft, percutaneous endoscopic gastrostomy (PEG) tube, and an automatic implantable cardioverter-defibrillator. There were no pertinent positives on review of systems. The patient denied fever, chest pain, night sweats, nausea, vomiting, and diarrhea. The patient had a 40 pack-year smoking history and a beer per day.

The vascular surgical service was consulted and ordered a right lower extremity arterial and venous duplex ultrasound which showed a partially thrombosed pseudoaneurysm in the right groin and was measured to be 9.8 x 5.7 cm with a patent neck. The patient was subsequently taken to the operating room three days later to obtain a right lower extremity angiography. During the procedure, the neck of the pseudoaneurysm was found to be 8 mm and was deemed a high risk for thrombin injection. Also, past CTA results demonstrated a venous fistula component involving the pseudoaneurysm, which prompted consideration for an open pseudoaneurysm repair. Due to the patient’s comorbidities of congestive heart failure (CHF), right-sided heart failure, and pulmonary hypertension, the possibility of an open repair was ruled out.

Ultimately, it was decided to treat the right superficial femoral artery pseudoaneurysm with an endovascular covered stent repair. During the procedure, retrograde access via the left common femoral artery was obtained using ultrasound. The procedure showed the right superficial femoral artery to be diffusely diseased without flow-limiting lesions and retrograde flow from the right superficial femoral artery to the right common femoral vein. There was no filing of the pseudoaneurysm sac. A 6 mm x 5 cm covered stent was placed into the right superficial femoral artery. The stent was ballooned with a 6 mm x 40 mm balloon to ensure there was excellent runoff to the superficial femoral artery (SFA) without extravasation or connection to the right common femoral vein. Protamine sulfate was used to reverse heparinization, and hemostasis was achieved 20 minutes later. The patient did well after the operation and was subsequently discharged four days later back to the nursing home facility.

## Discussion

Femoral artery pseudoaneurysms have increased in incidence over the years as the percutaneous and endovascular catheterization procedures are used more often by various specialties [1,2,7,9]. In the past, the frequency of femoral artery pseudoaneurysms was reported to be 0.02%, after ultrasound-guided catheterization procedures [7]. More current studies have reported that pseudoaneurysms will form in 3.5% of patients receiving percutaneous interventional cardiologic procedures [1]. Overall, femoral artery pseudoaneurysms have a reported incidence of 0.1%-5.5% [6]. Although percutaneous and endovascular procedures are the most common causes for the development of femoral artery pseudoaneurysms, they can also be caused by trauma, anastomotic leakage, and infection [9]. In addition, there is a female predominance of femoral artery pseudoaneurysms, and risk factors include smoking, hypertension, and peripheral artery disease [9].

Pseudoaneurysms differ from true aneurysms based on their anatomical involvement of the blood vessel wall [1,9]. True aneurysms are bounded by all three layers of the blood vessel wall, while pseudoaneurysms tend to be bounded by less than all three layers of the blood vessel wall [1,9]. True aneurysms mainly occur in the aorta and iliac vessels [9], whereas pseudoaneurysms are more likely to occur in the femoral, popliteal, and brachial arteries [9]. Femoral artery pseudoaneurysms occur most commonly beyond the bifurcation of the common femoral artery into the deep and superficial femoral arteries [1] because the common femoral artery is anterior to the head of the femur and the superior pubic ramus, both of which can cause arterial compression after catheter removal [1].

Patients with pseudoaneurysms usually present with a pulsatile mass that has a palpable thrill and a to-and-fro murmur [1,9]. Once suspected, arterial duplex ultrasound is currently the modality of choice [1]. The imaging study demonstrates the blood flow velocity, and it is less invasive, less expensive, and does not require the use of contrast agents [1,9]. In addition, duplex ultrasound has been shown to have a 94% sensitivity and a 97% specificity [9]. They provide excellent images of the size of the pseudoaneurysm [9]. This modality is also very efficient at establishing a relationship with other vessels adjacent to the pseudoaneurysm [9]. Other diagnostic modalities that can be used as well are digital subtraction angiography, CT scan, and MRI [9]. The risk associated with pseudoaneurysms includes bleeding, dissection, infection, thromboembolism, arteriovenous fistula, and limb ischemia [1,8].

Typically, a femoral artery pseudoaneurysm less than 2-3 cm tends to spontaneously thrombose and does not require interventional treatment [1,4,5,9]. However, those greater than 2-3 cm require intervention [1,4,5,9]. There are several different treatment modalities that are used and widely accepted [1,4,5,9]. Over the last decade, ultrasound-guided thrombin injection has become the primary treatment option in treating patients with pseudoaneurysms [6,9]. It is effective in patients already receiving anticoagulation, requires a very short time of treatment [6,9], typically is performed under local anesthesia, is less expensive, and can be used on noncompressible pseudoaneurysms [2,5,10]. Many studies have shown ultrasound-guided thrombin injection to be more efficient than ultrasound-guided compression with respect to time of the procedure and recurrence rate [4,5].

Furthermore, complications of ultrasound-guided thrombin injection include a risk that the injected thrombin could migrate beyond the neck of the pseudoaneurysm and get into the bloodstream causing thrombus and potential limb ischemia [10]. Also, patients can develop antibodies to the bovine thrombin used in the procedure and have an anaphylactic immunological response from the treatment [2,3,5,6,9]. This has been observed in patients who previously had thrombin injection for a pseudoaneurysm, then had a recurrence, and were retreated with thrombin injection [5,6,9].

Another option has been ultrasound-guided compression therapy. It has been shown to have good success rates, but the time to achieve thrombosis varies greatly. Some cases experienced a short interval of 30 minutes, while others experienced a prolonged time interval of up to four hours [1,2,4]. Ultrasound-guided compression achieves thromboses within the pseudoaneurysm by causing stasis leading to thrombosis within the cavity [7]. Also, this therapy is less effective in treating patients receiving anticoagulation and is contraindicated in patients with a noncompressible pseudoaneurysm [1,2,10]. Other disadvantages of this treatment are patient discomfort, prolonged procedure time, and a higher recurrence rate [4]. Some sources say that the failure rate in ultrasound-guided compression therapy for pseudoaneurysms has been as high as 41%-89% in patients receiving anticoagulant therapy [5]. Overall, the literature indicates that ultrasound-guided compression of a femoral artery pseudoaneurysm is a good alternative but not superior to ultrasound-guided thrombin injection [9,10].

Before ultrasound-guided thrombin injections and compression were discovered, the primary option for patients with femoral artery pseudoaneurysms was open surgical repair. It is a well-recognized and accepted treatment option but can be a problem in patients with limited cardiac reserve and significant comorbidities, like in our patient [1,16-20]. Over the years, this technique has been used less due to the advancement of less invasive treatments for pseudoaneurysm repair, but it is still a good alternative option. Indications for open surgical repair include hemodynamic instability, rapidly expanding groin hematoma, arteriovenous fistula, and severe infection. Overall, this method is very safe but is invasive, with a longer recovery time and a higher risk of complications than other methods [18-20]. Studies have found that the most common complications after open surgical repair of femoral pseudoaneurysms were bleeding, wound infections, and atrial fibrillation [15]. Older patients were shown to have a much longer hospital stay [15]. The patients with chronic obstructive pulmonary disease were found to be associated with a higher risk of wound infection and bleeding during the postoperative period [15].

Although not as well studied nor routinely performed, endovascular stent repair is also a treatment option for patients with femoral artery pseudoaneurysms [9]. Many small studies and case reports have shown this treatment to be effective in patients who are deemed high risk for a surgical operation [16-20]. Endovascular stent repair for femoral pseudoaneurysms can be performed under local anesthesia [13]. Overall, the literature agrees that in patients with multiple comorbidities or in hemodynamically unstable acute trauma patients, it can be a strong alternative to open surgical repair [13,14,16-20]. Thalhammer et al. had a very good technical success rate of 100% using endovascular covered stents in 16 cases of postcatheterization femoral artery pseudoaneurysms [14]. However, one patient underwent thromboendarterectomy for stent removal and four patients developed stent occlusion [13,14]. In a small study performed on similar patients, van Sambeek et al. found success in eight out of 10 patients using endovascular stent repair for femoral and popliteal artery pseudoaneurysms [11].

However, many questions still exist such as the patency rate of the stents, which has shown to fluctuate throughout the literature between 43% and 87% [9]. Covered stents have shown a much better one-year patency rate in this procedure [9]. Also, femoral artery pseudoaneurysms including superficial, deep, and common femoral arteries commonly occur near the hip joint where natural flexion and extension motions during walking occur which can cause kinking of the stent leading to turbulent flow of blood beyond it and causing thrombus formation [9,12,13]. Other complications include occlusion of side branches and intimal hyperplasia around the stent [13]. Overall, compared to the other methods of treatment for femoral artery pseudoaneurysms, endovascular stent treatment is significantly understudied and requires larger multicenter trials with stringent follow-up assessment [9,12]. But from what has currently been studied, it is a safe and effective technique that should be used to treat femoral artery pseudoaneurysm if all other options have been ruled out [13-20].

## Conclusions

Endovascular management of femoral artery pseudoaneurysms is a beneficial technique that can assist vascular surgeons in scenarios where the patient in question is at a very high surgical risk, has a large pseudoaneurysm at presentation, and has complicated anatomy and when other treatment options have failed. This technique has not been traditionally used for femoral artery pseudoaneurysm repair, but with the increasing usage of percutaneous, endovascular diagnostic, and interventional procedures among many specialties such as interventional radiology and cardiology, it has been used now. We anticipate that the incidence of femoral artery pseudoaneurysms will increase; thus, the complexity of cases will increase as well as the degree of difficulty in cases. We believe that endovascular repair of femoral artery pseudoaneurysms is a viable option in circumstances when no other options are available or indicated.
